# Understanding vulnerability to self-harm in times of economic hardship and austerity: a qualitative study

**DOI:** 10.1136/bmjopen-2015-010131

**Published:** 2016-02-17

**Authors:** M C Barnes, D Gunnell, R Davies, K Hawton, N Kapur, J Potokar, J L Donovan

**Affiliations:** 1 School of Social and Community Medicine, University of Bristol, Bristol UK; 2 Centre for Suicide Research, University of Oxford, Oxford, UK; 3 Centre for Suicide Prevention, University of Manchester, Manchester, UK

**Keywords:** QUALITATIVE RESEARCH, SOCIAL MEDICINE

## Abstract

**Objective:**

Self-harm and suicide increase in times of economic recession, but little is known about why people self-harm when in financial difficulty, and in what circumstances self-harm occurs. This study aimed to understand events and experiences leading to the episode of self-harm and to identify opportunities for prevention or mitigation of distress.

**Setting:**

Participants’ homes or university rooms.

**Participants:**

19 people who had attended hospital following self-harm in two UK cities and who specifically cited job loss, economic hardship or the impact of austerity measures as a causal or contributory factor.

**Primary and secondary outcome measures:**

Semistructured, in-depth interviews. Interviews were audio recorded, transcribed and analysed cross-sectionally and as case studies.

**Results:**

Study participants described experiences of severe economic hardship; being unable to find employment or losing jobs, debt, housing problems and benefit sanctions. In many cases problems accumulated and felt unresolvable. For others an event, such as a call from a debt collector or benefit change triggered the self-harm. Participants also reported other current or past difficulties, including abuse, neglect, bullying, domestic violence, mental health problems, relationship difficulties, bereavements and low self-esteem. These contributed to their sense of despair and worthlessness and increased their vulnerability to self-harm. Participants struggled to gain the practical help they felt they needed for their economic difficulties or therapeutic support that might have helped with their other co-existing or historically damaging experiences.

**Conclusions:**

Economic hardships resulting from the recession and austerity measures accumulated or acted as a ‘final straw’ to trigger self-harm, often in the context of co-existing or historically damaging life-experiences. Interventions to mitigate these effects should include providing practical advice about economic issues before difficulties become insurmountable and providing appropriate psychosocial support for vulnerable individuals.

**Strengths and limitations of this study**

- This is the first UK study of self-harm among people experiencing economic or austerity-related difficulties.
- Care was taken in sampling a wide range of characteristics including young men who are hard to reach and more likely to self-harm during recessions.
- The study provides insights about the experience of people who self-harmed because of financial or other economic difficulties.
- The sample was relatively small and participants were drawn from just two localities.

## Background

Periods of economic recession are usually characterised by rises in unemployment, increased levels of debt, bankruptcy and home repossessions. They are often accompanied by increases in the incidence of suicide.^1^ ^2^ Evidence about the impact of the 2008 global economic recession on suicide has been steadily growing, with studies showing that rates of suicide have increased, particularly among men and in countries and areas with the highest rises in unemployment.^3–5^ Many countries have responded to the recession with fiscal austerity measures and there is population-level evidence of the deleterious impact of these on health and suicide rates in Europe,^6^ and particularly in Greece.^7^

The bidirectional association of debt and unemployment with mental illness is well established. Debt and unemployment increase the risk of mental illness and suicide and those who are vulnerable to mental illness are at increased risk of job loss.^8^ Combinations of factors may be particularly damaging.^9^ ^10^ Debt and employment problems were mentioned as contributors in nearly a quarter of male suicides identified in a study of suicide deaths in Wales^11^ and in 13% of suicide deaths among men and women in a study carried out in England in 2010–2011.^12^

Those most at risk of job loss during periods of recession tend to have pre-existing mental health problems.^10^ ^13^ While it is clear that the poorest in society are likely to be hit hardest by economic downturns and their impact on mental health and suicide,^14^ the specific effects vary from country to country because of international differences in the degree to which economies are affected and the ways in which their social welfare policies and responses to recession mitigate effects.^2^ ^15^

There have been calls for more research to develop better understanding of the factors involved in economic recession and suicide,^16^ as there is little detailed information at the individual level on the impact of economic difficulties and austerity measures, and how these affect suicide risk.^9^ Descriptions of the effects of particular austerity measures on communities have indicated that the most vulnerable sections of the population are most affected by welfare changes and government cuts to services.^17^ ^18^

The aim of the study was to explore the events and experiences of people who self-harmed citing job loss, economic hardship or the impact of austerity measures as a contributory factor—to facilitate an understanding of the issues that triggered self-harm, the context of the difficulties, and to try to identify opportunities for the development of interventions to reduce or mitigate distress leading to self-harm and suicide.

## Methods

Study participants were sampled from patients who had self-harmed and attended hospital accident and emergency departments in two UK cities between December 2012 and March 2014, and were referred to a mental health specialist for a psychosocial assessment. The two UK cities had experienced large (60%) rises in unemployment during the economic recession between 2007 and 2012 (https:/http://www.nomisweb.co.uk). A number of austerity measures were implemented by the UK Government before and during the period of recruitment. Participants were approached for inclusion in the study if they had indicated that a precipitating factor in their self-harm included financial, employment or other difficulties related to economic hardship or austerity, such as housing problems or benefit changes. Sampling of participants was purposive, aiming to include individuals across the age range over 18 years, gender, employment status, social class and previous psychiatric illness. Patients who were identified as having overt psychotic symptoms at the time of hospital admission or who were unable to give informed consent, including those not fluent in English, were excluded.

Study participants were recruited in two ways: (A) at the time of their psychosocial assessment, or (B) following a retrospective review of the hospital self-harm databases which record information on the circumstances contributing to the episode; people who had self-harmed in the previous 6 months were recruited in this way. Approaches for recruitment were made only after assurance from care coordinators or GPs about the appropriateness of contact and that patients had agreed to a telephone call from the researcher about the study.

### Interviews

Interviews took place at a time and place convenient to participants, usually their home, National Health Service (NHS) or university rooms. Written consent was obtained for the study and to audiotape the interviews. The researcher completed the Suicide Intent Scale^19^ for each participant; values of 0–6, 7–12, 13–20 and >20 have been used in some previous studies to indicate low, moderate, high and very high suicidal intent, respectively.^20^ All participants were also monitored for distress preinterview and postinterview using a self-completed visual analogue scale.^21^ The research team have experience of interviewing suicidal individuals and have procedures to safeguard patient well-being. Findings suggest individuals are more likely to derive benefit from participation than experience harm.^21^

The interview topic guide was developed in collaboration with the clinical teams and four service-user advisors who had experienced mental illness, self-harm, job loss and financial difficulties. Interviews explored participants’ narratives leading up to the self-harm episode, including their perceptions and experiences of economic difficulties and the impact of austerity measures, their reactions to these, the timing of self-harm in relation to their difficulties, and the support they felt they needed. All interviews were conducted by MB, took approximately 1–2 h and were fully transcribed.

### Analysis

Data collection and analysis occurred concurrently and iteratively, cross-sectionally and in case-studies, according to the constant comparison methods of grounded theory.^22^ Data relating to the first four interviews were analysed by detailed scrutiny of the transcripts to identify common themes which were then coded (MCB) with the aid of NViVo software. A coding comparison exercise then took place with JLD and DG, with the service user team also reviewing some transcripts. Codes were refined and the framework used to code further ‘sets’ of transcripts within the sample: older men (range 35–54), older women (range 40–56), younger men (range 19–31) and younger women (range 23–37). Data were examined for similarities and differences within themes and across sets. Summaries of the case-studies of each participant were also made and considered for similarities and differences. Sampling continued until new themes no longer emerged from the data (saturation).

## Results

### Participant characteristics

Nineteen people aged 19–56 years were interviewed, including 9 men and 10 women (for details of participant characteristics and circumstances see table 1). The median score on the Beck suicide intent scales was 15 (range 10–23). Previous self-harm was common (n=14), with seven having self-harmed previously citing economic difficulties, and eight had been under the care of specialist psychiatric services in the past. Seven participants were clear that they had wanted to die when they self-harmed; the others described a mixture of intents—to end their despair, communicate distress, escape feelings of worthlessness, or punish themselves.

**Table 1:** Demographic and clinical characteristics of study participants

Characteristic	No participants
Gender	
Men	9
Women	10
Age group	
Under 30	5
31–40	5
41–50	5
51–60	4
Employment status	
Employed	6
Unemployed: receiving ESA*	7
Unemployed: receiving JSA†	5
Carer	1
Living with	
Family (1+ member of immediate family)	6
Partner	6
Alone	7
Participants with children	
Yes	8
No	11
Type of housing	
Social housing‡	6
Private rental	5
Lives with parents	4
Owns home /paying mortgage	3
Hostel	1
Suicide Intent Scores	
0–6 (low intent)	0
7–12 (moderate intent)	9
13–20 (high intent)	8
>20 (very high intent)	2
Previous self-harm	
Yes	14
No	5

*Employment Support Allowance (ESA) is a benefit for people unable to work due to disability or ill-health.

†Job Seekers Allowance (JSA) is a benefit for people actively looking for work.

‡Low-cost accommodation provided for people on low incomes.

### Emergent themes

Participants described a wide range of issues and difficulties as they told their stories about the self-harm episode and their experiences leading up to it. These interviews produced complex and distressing accounts of financial hardships interwoven with other past and present difficulties. The data were analysed cross-sectionally to identify key triggers of self-harm and similarities and differences in perceptions and experiences, and as case-studies to retain the uniqueness of the particular issues reported by the participants. Repeated comparison of cross-sectional and individual narrative data led to the insights presented here. The main findings relate to the details of and interplay between recession-related issues and past and current vulnerabilities, with a link to other major findings concerned with experiences of services and unmet need for help (details will be presented elsewhere). All names have been changed to protect the anonymity of the participants.

#### Employment, economic and austerity issues leading to the episode of self-harm

The first theme relates to the circumstances that led to the self-harm episode. Issues identified by participants as particularly distressing included losing or being unable to find work, fears or experiences of benefit changes or sanctions, increasing debt and housing difficulties such as the threat of eviction.

##### Employment difficulties

The range of participants’ employment states is shown in table 1. Six were in active work, with the remainder on benefits of some sort. The loss of work was a difficult experience, particularly where jobs had previously been relatively easy to obtain and where work was the ‘norm’. Those who lost jobs were deeply affected and expressed feelings of hurt, confusion and increasing despair.

I've always worked and I've always, you know, and its just- (pause) It's just all gone. (pause) … I've been through bad stuff before but I've coped with it and I've come through it and something's turned up but it just didn't happen this time. It still hasn't happened and you start thinking ‘what am I doing wrong, why is this happening?’ and I don't understand what I'm doing wrong. (Zoe 40)

Unfortunately the pub I took on closed down because it wasn't making enough money and since then it's just been a bit of me falling apart piece by piece. I enjoyed working and obviously—I'm a pretty social person, well I used to be anyway, and so I used to like seeing people and talking to people and that all went and I got left to it and, yeah, just became more and more of a recluse I guess, bit of a hermit, don't go anywhere, don't go out. (Joe, aged 31)

For the younger men and women difficulties started with being unable to find work after leaving school. The repeated experience of applying for jobs or apprenticeships but receiving no responses at all from potential employers was perceived particularly harshly. The lack of hope for the future because of these experiences and the lack of jobs, even for those with degrees or further training, led to diminished self-esteem and sometimes resort to potentially damaging coping strategies, including self-harm.

I had aspirations and stuff when I left school. I wanted to be an electrician. I went and did the courses and the rest of it and I applied for every apprenticeship within thirty miles of my house but ever since I was sixteen I've not even had one interview, not even a phone call or email back—no-one. …. I felt like I was stuck in a rut and the drugs and the alcohol…I ended up feeling more and more worthless every time you get shot down. There's only so many times you can be defeated before you start to defeat yourself and eventually I think I just got to that point where I'd had enough. (Paul, 23 years old)

[I would do] anything to do with retail, warehousing, painting, shelf stacking, bar staff, anything. No-one can (pause) be [bothered] to get back to me and say ‘very sorry’. Even if its ‘sorry, you're not successful’, that's fine but it's just no, they don't write back to you and it's just making me feel like—‘well alright then, I'm useless, there's no point in me even trying for a job ‘cause I'm never going to get one… (Ash, 19 years old)

So many people have a degree now that you have to have one but at the same time it's worthless ‘cause everyone has it so you just feel really inadequate … and its really disheartening when you get rejected…I just thought ‘I can't do this, this is too much, even if I get the job I won't be able to do it, I can't handle any of this, it's just too much’ and then I just really didn't want to live so I took an overdose. (Ellie 23)

##### Debt and benefits

Most (16) of the participants reported being in debt and having extremely restricted budgets. They talked in detail about the difficulty of managing day-to-day essentials and their fears about not being able to pay important bills such as rent and electricity. With restricted incomes, debts were difficult to manage and often quickly spiralled out of control. Many were fearful about how they would repay creditors and often they were not clear to whom they owed money as the debt had been passed on to other debt collection agencies. Missed payments led to fears that banks, bailiffs and organisations would take away the few possessions they had or threaten their homes. Bank loans that were manageable when individuals were employed could very quickly mount up, and student loans became a burden that with hindsight were not felt to be worth it. With little or no prospect of a solution to the financial difficulties, their distress mounted and sometimes led to self-harm.

We've got no [television] now and I've got no insurance and I got [water company] threatening to take me to court and put more money on top so I'm a hundred and something pound out there. I've got these different companies and I'm getting all confused now that I sold my debts so many times for my credit cards. I don't even know what they're writing to me about now. (Jane 48)

Finances—that was the ultimate trigger. Obviously I was feeling low anyway and I missed- I had forgotten to make a [council tax] payment [and they put me] onto a bailiff. It was just a genuine slip, a genuine slip and they were threatening to come and take our belongings, which they didn't do—as it turns out they were trying to scare me but that tipped me over the edge. (John 35)

I probably wouldn't have gone to uni. Probably would have chosen to just get a job and work my way up in a company ideally and get experience, um, and try not to be in debt…‘cause obviously it's a huge amount of money and then it just doesn't feel worth it at all. (Ellie 23)

I looked at my suicide note afterwards… Most of it was about the bank (pause) so anyway, yeah, the [benefits] I get tomorrow will go into the overdraft. I literally- I can't phone them because my phone's been cut off and I just- I cannot face going into the branch… I don't know what's going to happen now, I really don't. (Zoe 40)

The majority of participants were receiving benefits (table 1). The process of obtaining them was usually described as difficult or protracted, and continuing to receive them had also become a source of anxiety. At the time of the study, considerable changes to benefits were being made in the UK: the introduction of extra payments for ‘unused’ rooms in public housing (nicknamed the ‘bedroom tax’), the work capability assessment (introduced to determine access to benefits for sick and disabled adults) and benefit sanctions (the stopping of all benefits for a period of time following a failure to comply with particular rules). Fears about benefit changes preyed on respondents’ minds. It was these fears, as well as experiences of lost income, risk to accommodation and spiralling debt from sanctions and benefit changes, that led to increasing despair and self-harm.

Apparently I was fit enough for work even though I was absolutely falling apart, couldn't breathe, couldn't do nothing, my head was teetered and just gone and I wasn't there really and yeah, went to a tribunal, me money got squashed, got threw off and everything, no money for about six weeks and then I just snapped one day and ended up trying to overdose. (Joe 31)

The biggest thing and the biggest worry was when the bedroom tax came in…I've been worried sick since January… What's going to happen to my husband if I can't look after him (starts crying) and there's this thing and it's this thing with the bedroom tax that is cutting me to the core… I can wholeheartedly say it's definitely the situation with the bedroom tax that pushed me over the edge. (Jenny 56)

[When the benefit was sanctioned] that's when I went in the hospital…I had no money. I sold my watch and my rings in the pawnshops. … Tonight I don't have food … Bills to pay, electric to pay, gas, TV licence, and no money to pay them. (Bridget 50s)

### Housing difficulties

Debt and financial difficulties translated into housing difficulties for some. Two of three who owned their homes were being threatened with repossession, and several participants told stories of previous evictions or difficulties with benefits that threatened their homes:

Um, we were homeless…me losing my job affected our finances that bad we couldn't pay the rent ‘cause we were privately renting before and we couldn't afford to pay the rent so we got evicted. (John 35)

Yeah, well [the flat] is going to get repossessed I think. I've got to go to court in September but… if the benefits would have paid my interest for my mortgage then I wouldn't be in this situation… Now I find myself in the situation that it will get repossessed. So yeah, that's a bit scary ‘cause I'm not sure where I'm going to end up. (Maddie 38).

#### Co-existing or historical contextual vulnerabilities

The second theme comprised accounts of co-existing or long-standing problems that participants raised spontaneously when discussing their distress related to economic hardship. The focus of the interviews was on the financial and austerity issues that had led to their most recent episode of self-harm. However, in addition all participants also described other difficulties that contributed to their despair. These other factors seemed to become more salient as a source of despair as they described economic difficulties mounting or taking a sudden deleterious turn. At other times, these difficulties were identified as further justifications for their feelings of despair and worthlessness. Several of these difficulties were well known risk factors for self-harm. The vulnerabilities they described included abusive or neglectful childhoods, bullying, sexual identity issues, abusive adult relationships, significant bereavements and long-standing mental health problems.

… I've never liked my step-dad. Couldn't get on with him, he used to beat us up all the time and my mum's horrible so I left home when I was thirteen (Tommy 40 s)

I was bullied a lot, forced to stand up for myself and even then I was still getting beaten up by like twenty kids at a time. (Paul 23)

When I was being abused by the children's father … oh, it was like he was taking over my mind, he was taking over, kind of trying to strip me of everything… He was always criticizing how I was, um, and it was like psychological. There was physical and other abuse going on, but it was mainly psychological abuse where I got to the point where I thought ‘where's Jenny gone? Who am I now?’ (Jenny 56)

My granddad—well he passed away last year in March so obviously coming up to a year (pause) and he was a big part of my life so obviously really took it out of me to be honest. (Ash 19)

I think my sexuality's an issue as well. Not now it isn't, it was back when I was a lot younger (pause) and, um, I think that was probably when I first got depressed…I had real problems dealing with it so there's that and, um, the fact that I got diagnosed with HIV two years ago—I still haven't started dealing with that properly yet. (Matt 44)

I suffer with depression quite badly … I call it a lifelong thing… I've lost quite a few people in my time. Had bad things happen to me, um, don't think it's ever really been dealt with fully, um, so certain times it rears its ugly head and life isn't good…I think the whole self-harming was everything the money, the illness, lack of job. It was just [long pause] enough was enough. (Debbie 37)

As these quotations suggest, many of the participants had difficulties in addition to the economic hardship that they had identified as the trigger to their self-harm.

#### Perceptions of available help and support

The final emergent theme was a very large one concerned with participants’ experiences of forms of help, including health and social services, and their views about unmet needs. The details of these will be presented elsewhere. An important part of this theme, linked to the two above, was the participants’ clear expression that they needed clear practical help for their economic difficulties and counselling or some sort of therapeutic support for co-existing or historical problems.

It would have been nice to have some sort of counselling or I don't know, just someone to talk to about things and, you know, someone who could give me advice on where to go for certain issues. Like I say with the financial stuff it would have been nice for just rather than being told ‘well there's nothing that can be done about it’… (Lisa 23)

When you're feeling so low you can find it hard to access services so to be proactive and productive is too hard. Sometimes you need someone else to come and take control. (Ellie 23)

I think the biggest support that I could have done with and might still could do with is somebody who could fight our case for bedroom tax … An advocate, yeah, ‘cause I can't do it. I can't do it on my own, and obviously (husband) can't do it because he's stuck on the bed most of the time… Mentally I can't get my head round it any more. (Jenny 56)

These study participants had experienced economic difficulties that they indicated triggered self-harm. Several recognised that they needed help to access appropriate support for these and other pressing problems to avoid the risk of spiralling into despair.

## Discussion

The study participants described in detail their particular economic difficulties, mostly concerned with employment, debt and finances, benefit changes and housing problems—and the role they played in triggering the episode of self-harm that led to hospitalisation. Their economic problems often accumulated over time, such as repeatedly failing with job applications, increasing debts, and stringent budgeting, reaching a point where the difficulties felt insurmountable. In other cases, there was a ‘last straw’, for example imminent debt collection or benefit sanction. It was notable that fears about benefit changes, housing or job loss or meetings to discuss loans were at least as powerful as actual changes—and at times resulted in even greater anxiety. In addition to economic difficulties, the participants described a number of co-existing or earlier life experiences that troubled them. These included many of the established risk factors for suicide and self-harm—childhood neglect or abuse, bullying, domestic violence, bereavements, difficulties with sexual identity, and long-standing mental and physical health problems. Their accounts of financial and economic difficulties were interwoven with these. Study participants wanted clear, practical help for their economic difficulties and support for their other difficulties, but they did not know how to access it.

The findings from this study accord with a recent systematic review which reported strong associations between debt, depression, mental illness and suicide,^8^ and research indicating that unemployment is associated with increased risk of suicide.^16^ A clustering of economic strains including goal blockage, economic loss and anticipated economic strain has also been related to suicide.^9^ In the present study the narratives of the individuals illustrated how financial and other economic difficulties could accumulate to trigger self-harm, or combine with other co-existing or historical vulnerabilities to build an overwhelming sense of despair and worthlessness.

Individuals with poor mental health tend to be at increased risk of unemployment, and those who are unemployed are at greater risk of mental health problems.^10^ ^13^ Another well-recognised risk factor for self-harm is sexual abuse.^23^ This study has shown that such current and long-standing vulnerabilities may put individuals with economic difficulties at risk of self-harm in times of recession and austerity. This study cannot untangle whether it was the existence of other difficulties in addition to economic problems that increased their risk of self-harm, or whether when under economic strain these other current or historical vulnerabilities re-emerged and became more salient in contributing to their feelings of despair and low self-esteem. Nevertheless, an awareness of these issues could open up opportunities to develop interventions to support those with accumulations of difficulties, particularly in times of recession and austerity.

The recent recession has been associated with rises in unemployment and house repossessions^5^ as well as the introduction of a number of austerity measures by governments.^6^ These measures have been documented in detail.^18^ They have led to considerable difficulties for vulnerable individuals, with recent evidence of a link between the programme of reassessing people on disability benefits using the Work Capability Assessment and suicide.^24^ A qualitative study on the impact of the UK ‘bedroom tax’ showed that changes in welfare benefits in the UK led to worsening mental health and well-being.^17^ In this study we have shown that fears about the potential impact of austerity measures could trigger self-harm, in addition to actual changes in benefits. In comparison, increased investment, for example, in Scandinavian countries, may have prevented rises in suicide rates.^15^

Most of the participants in this study found support and practical advice about economic problems difficult to access. There is a particular need to identify the issues that affect men because they are most likely to die by suicide,^4^ and are a difficult group to access for research. The young men who participated in this study showed clearly that difficulties in finding work, especially the first job after completing education, seemed particularly troubling, as did the sense of low self-esteem and worthlessness created by the loss of aspiration and demoralisation from repeated fruitless applications. Previous research has reported long-term effects of experiencing unemployment after finishing education, including a high risk of being unemployed or working for reduced income 5 years later.^25^ Employers and Jobcentres should be aware of the potential damage that unresponsiveness to applications can cause, and the deleterious consequences of a policy in which making repeated job applications is a condition for receiving benefits, and the failure to meet application targets is punishable with benefit sanctions.

There is an increasing body of qualitative research aiming to understand aspects of self-harm and suicide,^26–28^ and generate ideas about interventions that can be developed. A study explored factors contributing to self-harm in the USA around the onset of the recent economic recession. The study focused on the period after the attempt, noting that participants reported positively about support they received afterwards that had not been available before and concluding that government funding should be increased during recessions to protect the most vulnerable from suicidal behaviour as a viable path towards immediate psychiatric treatment.^29^ This, and other studies, suggest that there could be opportunities to provide support earlier,^30^ an important factor given the continuing rise in self-harm during the study period.^31^

Study participants clearly stated their need for practical advice and help before debts and financial difficulties escalated into feelings of despair and hopelessness. This accords with a review of the suicide and recession literature suggesting that mental health professionals should routinely ask service users if they are in debt and direct them to sources of help or give extra support when negative financial events occur.^16^ This study also suggests that there is a need for employers and agencies such as debt collectors to have sensitivity about the consequences of their actions, particularly in times of recession and austerity when vulnerable people are at increased risk.

### Strengths and limitations

To the best of our knowledge this is the first UK study of self-harm among people experiencing economic or austerity-related difficulties. Given the widely reported difficulty in recruiting participants who have self-harmed these findings contribute understandings about the experiences of those who self-harm because of economic difficulties. A limitation of our sample is that it is relatively small and focused in two localities. Care was taken in sampling to include a wide range of characteristics, including some young men who are particularly hard to reach. While epidemiological evidence indicates that the rises in the incidence of self-harm and suicide during periods of recession occur largely in males,^4^ approximately half of the interviewees were female. This study does not claim to be representative—it aims to provide insights about the experiences of people who self-harmed because of financial or other economic difficulties that may be generalisable and assist in the development of interventions to help ameliorate distress and reduce suicide rates.

## Conclusions

Participants in this study described experiences of severe economic hardship, often in combination with other vulnerabilities, which led inexorably to episodes of self-harm, many with very serious intent. They had not been able to access effective advice for their economic difficulties or therapeutic support that might have helped with their other co-existing or historically damaging experiences before self-harming. These findings suggest some opportunities to develop interventions, such as providing practical advice about economic issues before difficulties become insurmountable, and targeting appropriate psychosocial therapy for those with co-existing and past vulnerabilities known to be risk factors for suicide and self-harm. After self-harm, a needs-based approach could be used to enhance support for people with combined financial and other difficulties. A navigator-style service could ensure access to financial advice after self-harm, through the maze of NHS and community services. As economic difficulties and austerity seem likely to continue, the development and robust evaluation of such initiatives is urgently required.

## References

[1] Oyesanya M, Lopez-Morinigo J, Dutta R Systematic review of suicide in economic recession. World J Psychiatry 2015;5:243–54. 10.5498/wjp.v5.i2.243 26110126 PMC4473496

[2] Stuckler D, Basu S, Suhrcke M The public health effect of economic crises and alternative policy responses in Europe: an empirical analysis. Lancet 2009;374:315–23. 10.1016/S0140-6736(09)61124-7 19589588

[3] Barr B, Taylor-Robinson D, Scott-Samuel A Suicides associated with the 2008–10 economic recession in England: time trend analysis. BMJ 2012;345:e5142 10.1136/bmj.e5142 22893569 PMC3419273

[4] Chang SS, Stuckler D, Yip P Impact of 2008 global economic crisis on suicide: time trend study in 54 countries. BMJ 2013;347:f5239 10.1136/bmj.f5239 24046155 PMC3776046

[5] Coope C, Gunnell G, Hollingworth W Suicide and the 2008 economic recession: who is most at risk? Trends in suicide rates in England and Wales 2001–2011*.* Soc Sci Med 2014;117:76–85. 10.1016/j.socscimed.2014.07.024 25054280 PMC4151136

[6] Quaglio G, Karapiperis T, Van Woense L Austerity and Health in Europe. Health Policy 2013;113:13–19. 10.1016/j.healthpol.2013.09.005 24176290

[7] Antonakakis N, Collins A The impact of fiscal austerity on suicide: on the empirics of a modern Greek tragedy. Soc Sci Med 2014;112:39–50. 10.1016/j.socscimed.2014.04.019 24788115

[8] Richardson T, Elliott P, Roberts R The relationship between personal unsecured debt and mental and physical health: a systematic review and meta-analysis. Clin Psychol Rev 2013;33:1148–62. 10.1016/j.cpr.2013.08.009 24121465

[9] Stack S, Wasserman I Economic strain and suicide risk: a qualitative analysis. Suicide Life Threat Behav 2007;37:103–12. 10.1521/suli.2007.37.1.103 17397284

[10] Butterworth P, Leach L, Pirkis J Poor mental health influences risk and duration of unemployment: a prospective study. Soc Psychiatry Psychiatr Epidemiol 2012;47:1013–21. 10.1007/s00127-011-0409-1 21681454

[11] Scourfield J, Fincham B, Langer S Sociological autopsy: an integrated approach to the study of suicide in men. Soc Sci Med 2012;74:466–73. 10.1016/j.socscimed.2010.01.054 20646811

[12] Coope C, Donovan J, Wilson C Characteristics of people dying by suicide after job loss, financial difficulties and other economic stressors during a period of recession (2010–2011): a review of coroners’ records. J Affect Disord 2015;183:98–105. 10.1016/j.jad.2015.04.045 26001669

[13] Lundin A, Lundberg I, Allebeck P Unemployment and suicide in the Stockholm population: a register-based study on 771,068 men and women. Public Health 2012;126:371–7. 10.1016/j.puhe.2012.01.020 22480712

[14] World Health Organisation. Impact of economic crises on mental health, 2011 http://www.euro.who.int/en/health-topics/noncommunicable-diseases/mental-health/publications/2011/impact-of-economic-crises-on-mental-health

[15] Norstrom T, Gronqvist H The Great Recession, unemployment and suicide. J Epidemiol Community Health 2015;69:110–16. 10.1136/jech-2014-204602 25339416 PMC4316842

[16] Haw C, Hawton K, Gunnell D Economic recession and suicide: a review of the literature. BMJ 2013;347:f5612 10.1136/bmj.f5612 24046157

[17] Moffatt S, Lawson S, Patterson R A qualitative study of the impact of the UK ‘bedroom tax’. J Public Health (Oxf) 2015. doi:10.1093/pubmed/fdv031 10.1093/pubmed/fdv031 PMC4894481 25774056

[18] O'Hara M Austerity Bites. Bristol, UK: Policy Press, 2014.

[19] Beck A, Schuyler D, Herman J Development of suicidal intent scales. In: Beck A, Resnick H, Lettieri D, eds. The prediction of suicide. Oxford: Charles Press, 1974:45–56.

[20] Hawton K, Casey D, Bale E Self-Harm in Oxford Annual Report 2012. Centre for Suicide Research and Oxford Health NHS Trust: Department of Health, 2012.

[21] Biddle L, Cooper J, Owen-Smith A Qualitative interviewing with vulnerable populations: individuals’ experiences of participating in suicide and self-harm based research. J Affect Disord 2013;145:356–62. 10.1016/j.jad.2012.08.024 23021191

[22] Glaser B, Strauss A The discovery of grounded theory. London: Weidenfeld & Nicholson, 1967.

[23] Joiner T, Sachs-Ericsson N, Wingate L Childhood physical and sexual abuse and lifetime number of suicide attempts: a persistent and theoretically important relationship. Behav Res Ther 2007;45:539–47. 10.1016/j.brat.2006.04.007 16765909

[24] Barr B, Taylor-Robinson D, Stuckler D ‘First, do no harm’: are disability assessments associated with adverse trends in mental health? A longitudinal ecological study. J Epidemiol Community Health; 2015. Published Online First: 16 Nov 2015. 10.1136/jech-2015-206209 PMC4819657 26573235

[25] Skans O Scarring effects of the first labour market experience: a sibling based analysis. Working Paper Series from IFAU Institute for Evaluation of Labour Market and Education Policy 2004:14 http://www.ifau.se/upload/pdf/se/2004/wp04–14.pdf

[26] Biddle L, Donovan J, Owen-Smith A Factors influencing the decision to use hanging as method of suicide: a qualitative study. Br J Gen Pract 2010;197:320–5. 10.1192/bjp.bp.109.076349 20884956

[27] Owens C, Lambert H, Donovan J A qualitative study of help seeking and primary care consultation prior to suicide. Br J Gen Pract 2005;55:503–9. 16004734 PMC1472785

[28] Hunter C, Chantler K, Kapur N Service user perspectives on psychosocial assessment following self-harm and its impact on further help-seeking: a qualitative study. J Affect Disord 2013;145:315–23. 10.1016/j.jad.2012.08.009 22925352

[29] Elliott M, Naphan D, Kohlenberg B Suicidal behaviour during economic hard times. Int J Soc Psychiatry 2015; 61:492–7. 10.1177/0020764014556391 25359733

[30] Hill K, Dallos R Young people's stories of self-harm: a narrative study. Clin Child Psychol Psychiatry 2012;17:459–75. 10.1177/1359104511423364 22104364

[31] Hawton K, Bergen H, Geulayov G Impact of the recent recession on self-harm: longitudinal ecological and patient-level investigation from the Multicentre Study of Self-harm in England. J Affect Disord 2016;191:132–8. 10.1016/j.jad.2015.11.001 26655123

